# Chitosan: An Overview of Its Properties and Applications

**DOI:** 10.3390/polym13193256

**Published:** 2021-09-24

**Authors:** Inmaculada Aranaz, Andrés R. Alcántara, Maria Concepción Civera, Concepción Arias, Begoña Elorza, Angeles Heras Caballero, Niuris Acosta

**Affiliations:** 1 Departmento de Química en Ciencias Farmacéuticas, Universidad Complutense de Madrid, 28040 Madrid, Spain; iaranaz@ucm.es (I.A.); andalcan@ucm.es (A.R.A.); mccivera@ucm.es (M.C.C.); carias@ucm.es (C.A.); belorza@ucm.es (B.E.); aheras@farm.ucm.es (A.H.C.); 2 Instituto Pluridisciplinar, Universidad Complutense de Madrid, Paseo Juan XXIII, n 1, 28040 Madrid, Spain

**Keywords:** chitosan, chitin, biological activity, drug delivery, antioxidant, antimicrobial, metallic nanoparticles, biocatalysis

## Abstract

Chitosan has garnered much interest due to its properties and possible applications. Every year the number of publications and patents based on this polymer increase. Chitosan exhibits poor solubility in neutral and basic media, limiting its use in such conditions. Another serious obstacle is directly related to its natural origin. Chitosan is not a single polymer with a defined structure but a family of molecules with differences in their composition, size, and monomer distribution. These properties have a fundamental effect on the biological and technological performance of the polymer. Moreover, some of the biological properties claimed are discrete. In this review, we discuss how chitosan chemistry can solve the problems related to its poor solubility and can boost the polymer properties. We focus on some of the main biological properties of chitosan and the relationship with the physicochemical properties of the polymer. Then, we review two polymer applications related to green processes: the use of chitosan in the green synthesis of metallic nanoparticles and its use as support for biocatalysts. Finally, we briefly describe how making use of the technological properties of chitosan makes it possible to develop a variety of systems for drug delivery.

## 1. Introduction

Chitin and its deacetylated derivative, chitosan, are a family of linear polysaccharides composed of varying amounts of (β1→4) linked residues of *N*-acetyl-2 amino-2-deoxy-D-glucose (glucosamine, GlcN) and 2-amino-2-deoxy-D-glucose (*N*-acetyl-glucosamine, GlcNAc) residues. Chitosan is soluble in aqueous acidic media via primary amine protonation. In contrast, in chitin, the number of acetylated residues is high enough to prevent the polymer for dissolving in aqueous acidic media.

Chitin is a very abundant biopolymer that can be found in the exoskeleton of crustacea, insect’s cuticles, algae and in the cell wall of fungi. Chitosan is less frequent in nature occurring in some fungi (*Mucoraceae*). Historically, commercial chitosan samples were mainly produced from chemical deacetylation of chitin from crustacean sources. More recently, chitosan from fungi is gaining interest in the market, driven by vegan demands. Moreover, these samples are better controlled in terms of low viscosity and exhibit a very high deacetylation degree [1]. Production from insect cuticles is also gaining interest, driven by the increased interest in protein production from these sources.

The interest in chitin and chitosan relies on the myriad biological and technological properties exhibited by these polymers (Table 1). However, these properties are tightly related to the physicochemical properties of the polymers (mainly molecular weight and acetylation degree) [2]. Therefore, when working with chitin and chitosan a good and completed polymer characterization is mandatory. Several methodologies have been described to characterize chitin, chitosan and chitooligosaccharides, a description of which is far from the objective of this paper—but for interested readers, we recommend publications [3,4].

Chitosan is the only polycation in nature and its charge density depends on the degree of acetylation and pH of the media. The solubility of the polymer depends on the acetylation degree and molecular weight. Chitosan oligomers are soluble over a wide pH range, from acidic to basic ones (i.e., physiological pH 7.4). On the contrary, chitosan samples with higher Mw are only soluble in acidic aqueous media even at high deacetylation degrees. This lack of solubility at neutral and basic pH has hindered the use of chitosan in some applications under neutral physiological conditions (i.e., pH 7.4). This is the reason why a great number of chitosan derivatives with enhanced solubility have been synthetized. 

In 2019, the global chitosan market size was valued at USD 6.8 billion, and it is expected to expand at a revenue based CAGR of 24.7% between 2020 and 2027. The drivers for the market’s growth are the increasing application of the polymer in water treatment and several high-value industries such as the pharmaceutical, biomedical, cosmetics and food industries [14]. Some of the interest areas identified include the modification of the polymers to extend their applicability; knowledge of the mechanisms involved in the biological activity of chitosan, chitosan derivatives and chitooligosaccharides; and the in-depth study of chitosanolytic and chitinolytic enzymes presented in different microorganisms [15].

This review aims to provide readers with a general overview of the state of the art of chitosan science, covering different aspects such as polymer chemistry, biological and technological properties and applications in drug delivery and as a biocatalyst.

## 2. Technological Chitosan Properties

### 2.1. Solubility

Chitosan is produced by deacetylation of chitin; in this process, some *N*-acetylglucosamine moieties are converted into glucosamine units. The presence of large amounts of protonated -NH_2_ groups on the chitosan structure accounts for its solubility in acid aqueous media since its pKa value is approximately 6.5 [16]. When around 50% of all amino groups are protonated, chitosan becomes soluble [17]. 

Chitosan solubility depends on different factors such as polymer molecular weight, degree of acetylation, pH, temperature, and polymer crystallinity. Homogeneous deacetylation (alkali treatment, 0 °C) of chitin permits the production of polymers soluble in aqueous acetic acid solutions with DD as low as 28%, with this value never being reached under heterogeneous deacetylation (alkali treatment, high temperatures). Moreover, with a DD of 49%, the samples are soluble in water. This behaviour is explained by the fact that homogeneous deacetylation leads to an increase in the number of glucosamine units and a modification in the crystalline structure of the polymer. Depending on polymer DD, these modifications range from a reduction in crystal size and crystal perfection to the presence of a new crystal structure close to β-chitin [18]. Sogias et al. [19] studied the role of crystallinity and inter- or intramolecular forces on chitosan solubility; in this work, a parent chitosan sample was half re-acetylated with anhydride acetic or fully *N*-deacetylated under homogeneous conditions. After reacetylation, the solubility of the polymer was expanded until pH 7.4, while a slight reduction in the solubility range of the fully deacetylated chitosan was determined. The lower solubility was explained due to the increase in the polymer crystallinity after deacetylation, which offsets the effect of the increase in glucosamine moieties. On the contrary, a reduction in the crystallinity was observed in the half-acetylated sample. The use of hydrogen bond disruptors such as urea or guanidine hydrochloride also alters the solubility window of chitosan. In fact, by a combination of chemical and physical disruption of the hydrogen bonds, broad solubility is achieved.

### 2.2. Viscosity

The viscosity of polymers is a parametre of great interest from the technological point of view since highly viscous solutions are difficult to manage. Moreover, viscometry is a powerful tool for determining chitosan’s molecular weight, as it is a simple and rapid method even though it is not an absolute method, therefore requiring the determination of constants that are specific to the solvent. The average molecular weight is determined by the Mark–Houwink–Sakurada equation, which relates this parametre with the intrinsic viscosity:

η = KM_v_^α^ (1)

where K and α are constants that must be determined experimentally. Several values of K and α have been reported depending on the solvent composition, pH, and ionic strength [20]. Chitosan viscosity depends on the molecular weight of the polymer and deacetylation degree and decreases as the molecular weight of chitosan is reduced. In fact, viscosity can be used to determine the stability of the polymer in solution, as a reduction is observed during polymer storage due to polymer degradation [21]. Shear viscosity increases with chitosan deacetylation degree. The shear viscosity at the same rate was studied in two samples with different deacetylation degrees (91% vs. 75%) and represented versus intrinsic viscosity [22]; it was reported that shear viscosity was larger for those samples with the highest deacetylation degree; when the curves were evaluated, straight lines were observed in both chitosan samples This is explained due to the nature of chitosan, as this polymer is a cationic polyelectrolyte because of the amine protonation in acidic media. Therefore, the higher the DD, the larger chain expansion is expected, as more glucosamine units are found in the polymer chain, leading to a greater charge density in this sample. In order to modulate chitosan viscosity, the addition of different co-solvents has been evaluated; in this sense, Kassai et al. [20] studied the effect of the addition of isopropanol and ethanol to a chitosan solution in 1% acetic acid, reporting that the presence of the cosolvents decreased the intrinsic viscosity of the polymer.

## 3. Chemistry of Chitosan

**Figure 1:**
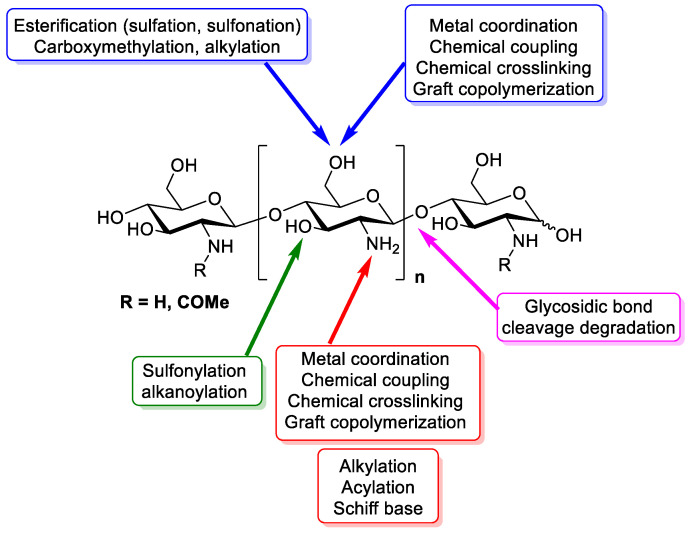
Functional groups in chitosan’s structure that are able to be chemically modified.

As seen in Figure 1, the reactive groups found in chitosan are a primary amino group (C2) and primary and secondary hydroxyl groups (C6, C3). Glycosidic bonds and the acetamide group can also be considered functional groups. These functional groups allow for a great number of modifications, producing polymers with new properties and behaviours.

Chitosan derivatives have been produced, aiming to improve chitosan’s properties, such as solubility or biodegradability, or to introduce new functions or properties. For instance, solubility has been improved in water aqueous media by deacetylation, depolymerization, or quaternisation among other processes [23]. New chitosan activities have been reported after its modification, for example, 6-O-sulphated chitosan promotes neuronal differentiation while phosphorylated chitosan inhibits corrosion [24,25].

**Figure 2:**
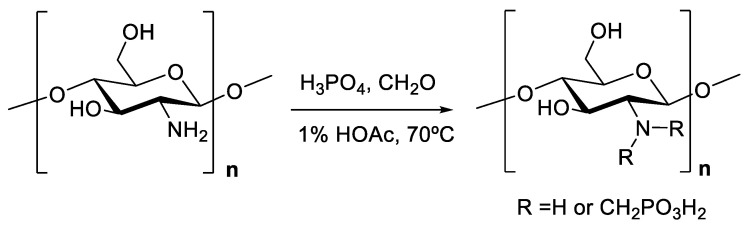
Scheme of phosphorylated chitosan derivative synthesis.

The field of chitosan chemistry is wide, and in this review, we want to focus on two types of processes, chitosan phosphorylation and chitosan degradation. Our group has participated in the development of a phosphorylated derivative via a simple method in which chitosan and phosphorus acid are mixed at the same ratio and formaldehyde is added at 70 °C [26] (Figure 2). 

This *N*-methylene phosphonic chitosan is soluble in water and keeps the filmogenic properties of the parent chitosan. With a similar methodology, a soluble in water *N*-methylenephenyl phosphonic chitosan has been produced [27]. Additionally, the surfactant derivative *N*-lauryl-*N*-methylene phosphonic chitosan was produced via *N*-alkylation of *N*-methylene phosphonic chitosan [28]. This derivative has a lower solubility in aqueous media compared to *N*-methylene phosphonic chitosan but better solubility in organic media and forms micelles. *N*-methylene phosphonic *N*-methylene carboxylic chitosan has been obtained in water-soluble form using *N*-methylene phosphonic chitosan and glyoxylic acid. The polymer maintains the filmogenic properties of parent chitosan and, because of the presence of multidentate ligands, its use as a bivalent metal chelating agent is proposed [29].

Although the use of chitosan as a gene carrier has been reported, the use of this biopolymer for this application is limited due to a relatively low transgenic efficacy. Phosphorylated derivatives have shown better performance (transfection was improved 100-fold) and therefore are more suitable than chitosan to this end. Moreover, phosphorylated derivatives also exhibit and improve metal ion chelating activity when compared to the parent chitosan [30,31].

Due to the presence of cleavage glycosidic bonds, it is possible to degrade chitosan, thus reducing its molecular weight. As previously mentioned, the control of chitosan depolymerization (polymer size) permits us to control some properties such as solubility or viscosity. Moreover, the biological and technological properties of chitosan are related to size, among other properties as previously reviewed [2]. Chitosan degradation can occur through different mechanisms such as acid hydrolysis, oxidative–reductive or nitrous acid depolymerization, ultrasonic degradation, or enzymatic degradation using specific and non-specific enzymes. Chitosan has four types of glycosidic linkages -D-D-, -A-A-, -A-D- and -D-A- (where A and D denote *N*-acetylglucosamine and glucosamine monomers, respectively). Depending on the process, there is a prevalence in the breakage of certain linkages and therefore different samples can be produced from the same parent chitosan by selecting different methodologies. Chemical and physical methods are less selective than enzymatic ones for producing specific patterns due to enzyme-specific recognition but by controlling the parametres of the process some control over the composition can be gained. 

Ultrasonic degradation of chitosan does not affect the degree of acetylation or polydispersity of the recovered polymers allowing for the moderate degradation of the polymer [32]. The rate of degradation depends on the acetylation degree of the parent chitosan and not on the initial molecular weight [33].

Hydrogen peroxide produces random degradation of chitosan in a faster manner than ultrasonic methodologies, producing a significant number of monomers and chitooligosaccharides, the composition of which depends on the temperature and H_2_O_2_ concentration [34]. Nitrous acid depolymerization can be considered somewhat specific since HNO_2_ attacks the primary amine in glucosamine and subsequently the cleavage of the glycosidic bonds occurs. That is, only the glycosidic linkage following a *D*-unit can be cleaved [35]. The chemical processes yield large amounts of monomers (*D*-glucosamine) and when the intended final products are chitooligosaccharides rather than low molecular weight chitosan, the yields are low [36]. HNO_2_ provokes the formation of 2,5-anhydro-*D*-mannose at the new reducing end, which may be considered a disadvantage of this acid. When chitosan is degraded by HCl, the polymer not only suffers the hydrolysis of the *O*-glycosidic linkage between residues but also the *N*-acetyl linkage can be hydrolyzed but at a lower rate. The hydrolysis rate of D-D and D-A glycosidic linkages is lower than the hydrolysis of A-A and A-D, therefore the reducing ends are dominated by acetylated units [37]. By using a controlled precipitation method with methanol, it has been possible to obtain chitooligosaccharides with DPs up to 16 with few low molecular weight oligomers with a good yield [38]. 

The specific enzymatic degradation of chitosan occurs with a family of enzymes named chitosanases (EC 3.2.1.132) and chitinases (EC 3.2.1.14). Chitosanases are glycosyl hydrolases that catalyse the *endo* hydrolysis of β-1,4-glycosidic bonds of partially acetylated chitosan to release chitosan oligosaccharides (COS) with little monomer release [39]. Chitosanase specifically hydrolyses chitosan by cleavage of glycosidic bonds with a -DD·DA- pattern or a -DD·DD-pattern. Chitinases, which occur in families GH18 and GH 19, are glycosyl hydrolases that can degrade both A-A and A-D linkages and show no activity against D-D linkages. Chitinases can be classified into two major categories (endochitinases and exochitinases) according to their mode of action [40]. 

Non-specific enzymes, also called promiscuous enzymes, are also able to degrade chitosan. These enzymes belong to the protease, lipase, cellulase, and hemicellulase families, among others. Lysozyme is one of the most studied due to its relationship with polymer biodegradation. this enzyme is a protease that hydrolyses chitosan by cleavage of glycosidic bonds with A-A-A-A- pattern or A-A-A-D-pattern, while A-D-A-pattern or D-D-A-A are not or very slowly hydrolysed by lysozyme [14,17]. Apart from the previously mentioned lysozyme, other proteolytic enzymes such as pepsin, papain and pronase caused chitosan depolymerization, rendering low molecular chitosans (4–10 kDa) as the main products and chitooligosaccharides and monomers in smaller amounts. Results indicated that papain and pepsin had a similar action pattern. Both enzymes decreased LMWC acetylation degree when compared to the parent chitosan; DP 2–6 were detected in the supernatant monomers (D and A) and oligomers. Pronase showed different behaviour since no glucosamine was detected. It showed selectivity through A-A and A-D, resulting in products having A monomers at the reducing end [41]. 

Neutral protease degraded chitosan in a manner dependent on the deacetylation degree. The higher the DD, the higher the Km and the lower the Vmax. During degradation, a reduction in the DD of the recovered LMW chitosans was observed. An analysis of the partially hydrolysed chitosan revealed that the enzyme degraded D-D and A-D β-1,4-glycosidic linkages, producing a mixture of hetero oligosaccharides carrying an A residue at the reducing end [42]. The same authors have studied the effect of the chitosan molecular weight in the enzymatic activity since this parametre affects its chain flexibility in solution, which in turn may affect its affinity for the enzyme in hydrolysis reactions. Their results showed a lower affinity of the enzyme with a slower degradation rate when high molecular weight chitosan samples were tested [43].

Hemicellulase, an enzyme related to the degradation of hemicellulose, has proven its ability to reduce chitosan molecular weight in a manner that depends on the deacetylation degree of the chitosan, rendering lower molecular weight samples when a chitosan sample with a DD of 85% was tested. Dimers, trimers, tetramers, pentamers and hexamers were observed after 4 hours of reaction, and the enzyme was considered endo-acting since no *N*-acetylglucosamine was detected [44].

Lipases have also proved their ability to hydrolysate chitosan, although the degradation rates are slower than the ones reported by other enzymes such as proteases or hemicellulose. Controlling reaction temperature, a commercial lipase rendered low molecular weight samples or chitooligosaccharides [45,46]. This lipase acted following both *exo* and *endo* cleavage mode. The presence of D end products indicates that it acted on chitosan in an *exo*-type mode while the sharp reduction in viscosity during the hydrolysis indicates that an *endo* splitting occurred in the initial hydrolysis stage. Therefore, by controlling the reaction time the final products can be led to oligomers with high DP or monomers. The polymer polydispersity depended on the used enzyme, lipase from wheat germ rendered samples with very wide molecular weight while lipase from *R. japonicus* exhibited better control over polydispersity [47].

The data previously showed that it is possible to somehow select the degradation products (LMW chitosans or oligosaccharides) by selecting the appropriate methodology (Table 2). As we can see in some sections of this review, specific biological and technological behaviour of chitosan degradation products depends not only on the method (physical, chemical, or enzymatic) selected to degrade the chitosan but also on the type of chemical or enzyme used for these processes. This effect is more related to the degraded polymer pattern rather than to the size or acetylation degree of the samples.

## 4. Biological Properties

Chitin, chitosan, oligosaccharides, and derivatives exert many biological activities including antitumoral, antimicrobial, antioxidant, and anti-inflammatory activities, which could be used as therapeutic polymers. It is remarkable that up today chitosan and chitosan hydrochloride are only accepted as excipients by the regulatory agencies and not as a drug for the treatment of diseases.

### 4.1. Antimicrobial Activity

Bacterial resistance to antibiotics is a critical public health concern and, therefore, there is an urgency to find alternatives to antibiotics. Chitosan, chitosan derivatives and chitooligosaccharides exert antimicrobial activity against different microorganisms, including bacteria, filamentous fungi, and yeast [48]; some examples of the different microorganisms sensible to chitosan are shown in Table 3. Chitosan seems to have a growth-inhibitory activity since bacteria is able to grow after the polymer is removed from the media. This is of importance since resistant populations might emerge if the cells adapt to chitosan [49].

Due to chitosan’s poor solubility above pH 6.5, the use of chitooligosaccharides is under consideration as polycationic biocides since they are soluble in water. Chitosan soluble derivatives such as sulphated chitosan, *N*-trimethyl chitosan, *N*-diethylmethyl chitosan or 2,6-diamino chitosan also avoid the use of acidic environments and exert antimicrobial activity [63,64,65]. This antimicrobial activity has applications in different fields such as the food, textile, or cosmetic industry, among others. Thus, due to the ability of chitosan to form shift bases, some new chitosan derivatives based on heterocyclic moieties have been developed, including pyrazole ring and furanyl, pyridyl, or thiophenyl moieties. Although these derivatives do not show higher solubility in aqueous media, their performance against gram-positive microorganism was improved when compared with the parent chitosan [66]. 

How these polymers (chitosan, chitooligosaccharides and derivatives) exert their antimicrobial activity is still under discussion. This fact can be explained by taking into account the lack of appropriate polymer characterization, purity issues, the use of different microorganisms, and the lack of methodological uniformity. Some studies point to the reduction in cell membrane permeability due to polymer coating on the surface of the cells that blocks cell access to nutrients. This process occurs due to the interaction of -NH_2_ groups from chitosan chains with -COO- groups on the external cell membranes of microorganisms. Therefore, the antimicrobial activity depends on the acetylation degree. It has also been hypothesized that chitosan can penetrate the cells and block RNA transcription as a result of adsorption with bacterial DNA [9]. Most likely, these mechanisms are not mutually exclusive, and several events are related to cell growth inhibition.

Intrinsic factors affecting the antimicrobial chitosan activity are due to the polymer characteristics such as Mw, acetylation degree, polymer viscosity, or polymer concentration. The solvent used to dissolve the polymer also affects its behaviour. We have observed that typical solvents used to dissolve chitosan such as acetic acid, citric acid, or buffers such as AcOH-NaAc exert some antimicrobial activity per se (unpublished results). Other factors with great impact on the antimicrobial activity are related to the tested microorganism, growth media, pH, temperature, ionic strength, or physiological state of the cells. 

The effect of polymer size is controversial. Some studies claim that the antimicrobial activity of chitosan improves with the polymer size and have found that oligosaccharides have lower antimicrobial activity [67,68,69]. When comparing chitooligosaccharides, those showing higher DP exhibited higher antimicrobial activity [70]. Moreover, Tokura and co-workers reported that chemically produced chitooligosaccharides of 2200 Da not only had no antimicrobial activity but also served as growth accelerators of E. coli, while a sample with 9300 Da inhibited bacterial growth [71]. On the contrary, other studies showed better antimicrobial activity for a lower molecular weight chitosan sample (55 kDa) than a higher one (155 kDa); in the same study when a sample of 90 kDa was tested a promotion of bacterial growth was observed [72]. In another study, different tendencies were observed depending on the pH of the media. In acidic pH conditions, the antimicrobial activity increased with increasing MW. However, at neutral pH, antimicrobial activity increased as the MW decreased [73]. Even so, no trend on the effect of chitosan Mw on antimicrobial activity has been reported [74]. Regarding acetylation degree, it seems that the lower the acetylation degree, the better the antimicrobial activity [69,74,75].

After depolimerization of a chitosan sample (400 kDa, DD~85%) with hemicellulose, a set of chitosan samples with similar DD and Mw ranging from 130 to 2.8 kDa and a chitooligosacharide sample with Mw 1.4 were produced. Some of these samples were also half-acetylated, furnishing two chitosan samples with Mw of 53 and 18 kDa, and some chitoligosaccharides with Mw of 1.4 kDa. Both chitooligosacharides samples and the half-acetylated samples were water soluble, while the others were not soluble in water. All samples were tested against Staphylococcus aureus, Escherichia coli, and Candida albicans. In this study, water soluble chitosans and oligosaccharides did not exhibit antimicrobial activity; in fact, they promoted the growth of C. albicans. Insoluble chitosan samples exhibited antimicrobial activity with the most pronounced effect when medium molecular weight samples were tested (Mw 78–48 kDa) [76].

Our group has studied the antimicrobial activity of low molecular weight chitosans and oligosaccharides produced by enzymatic degradation in order to determine if the polymer pattern has some effect on this activity. Chitooligosaccharides were produced by two different processes; thus, in process P1 chitosan was enzymatically depolymerized with chitosanase, while in process P2 the sample was depolymerized in a two-step process with HNO_2_ and chitosanase. The samples were tested against E coli and L. monocytogenes. COS from P1 showed a higher capability to inhibit bacterial growth than COS from P2. In both cases, COS were more effective at inhibiting E. coli (Gram-negative) than the Gram-positive L. monocytogenes. Antimicrobial activity depended on the production process and composition and structure of COS. COS produced in a one-step enzymatic procedure showed better antimicrobial activity than those produced in the two-step chemical–enzymatic process even when the samples exhibited similar DA and MW [77].

### 4.2. Antioxidant Activity

Antioxidants are gaining interest due to the relationship between oxidative stress and several diseases such as Alzheimer’s disease, Parkinson’s disease, Huntington’s disease, amyotrophic lateral sclerosis, and cancer. Moreover, it is related to complications in other diseases such as diabetes [78,79,80]. 

Chitosan contains an amino and several hydroxyl groups, which can react with free radicals exhibiting scavenging ability. Some chitosan derivatives such as chitosan sulphates or N-2 carboxyethyl chitosan exhibited improved antioxidant activity [81,82,83]. Chitooligosaccharides have also been chemically modified to improve their antioxidant activity, for instance by modification of the polymers with gallic acid [84,85] or phenolic compounds [86]. 

**Figure 3:**
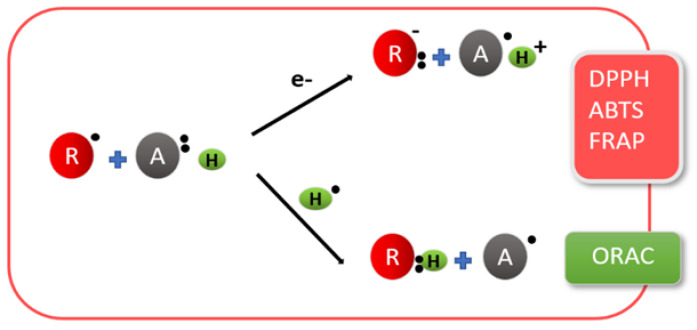
Methodologies used to determine antioxidant activities.

Different methodologies have been used to determine chitosan and its derivatives’ antioxidant assays, which includes DPPH (2,2-diphenyl-1-picryl-hydrazyl-hydrate), ABTS (2,2-azinobis (3-ethylbenzothiazoline-6-sulphonic acid), and FRAP (ferric antioxidant power) assays, peroxide and hydroxyl radical scavenging assays or the use of macrophage models. DPPH and ABTS assays are based on electron and H atom transfer, while the FRAP assay is based on electron transfer reaction, as depicted in Figure 3. The ORAC (oxygen radical absorbance capacity assay) is also widely used to test antioxidant activities.

The disparity between the polymers tested and the methodologies used to test the activity produces considerable differences in the polymer concentrations that range from 50 µg/mL to 400 mg/mL [83]. Antioxidant activity is more remarkable for low molecular weight samples rather than for high molecular weight ones since shorter chains form fewer intramolecular hydrogen bonds and therefore the reactive groups are more accessible, contributing to the radical scavenging activity [87,88]. Regarding the effect of the acetylation degree, the antioxidant activity seems to decrease when this parametre increases [88]. 

### 4.3. Anti-Inflammatory Properties

The inflammatory process is an automatic physiological response of the body related to tissue damage. The main goal of the inflammatory response is to bring circulating leukocytes and plasma proteins to the site of the infection or tissue damage, to eliminate the causative agent, when possible, and to start the healing process. Although inflammation is necessary for survival, when it is very severe, unable to eradicate the causative agent, or is directed against the host, the inflammatory process may cause damage. The inflammatory process is strongly related to the generation of free radicals. Again, this activity seems to be more remarkable when the molecular weight of the chitosan is reduced and chitooligosaccharides exhibit higher activity. 

After chitosan (300 kDa) depolymerization with cellulose, the activity of degraded polymers with medium molecular weight, low molecular weight and chitooligosaccharides (156, 72, 7 and 3.3 kDa) were tested in terms of NO secretion, cytokine production, and mitogen-activated protein kinase pathways in a model of lipopolysaccharide (LPS)-induced murine RAW 264.7 macrophages. Chitosan samples (parent, medium, and low) significantly inhibited NO production. On the contrary, the opposite effect was observed with the COS. The mechanism followed by the medium and low Mw chitosan to inhibited NF-κB activation and iNOS expression differed. For medium chitosan (156 kDa) the process occurred via the binding to CR3 while for low molecular weight chitosan the process occurred via the binding to CR3 and TLR4 receptors. On the contrary, the lower molecular weight chitosans activated NF-κB and enhanced iNOS expression by binding to CD14, TLR4, and CR3 receptors to activate JNK signalling proteins [89]. In general, chitooligosaccharides are studied in more detail for this application compared to chitosan, due to their better solubility in aqueous media and better performance. 

The effect of acetylation degree on the anti-inflammatory activities of COS has also been studied. Chitooligosaccharides with MW between 0.2 and 1.2 kDa were enzymatically depolymerized, depending on the enzyme, fully deacetylated (fdCOS, mainly GlcN, (GlcN)2, (GlcN)3, and (GlcN)4), partially acetylated (paCOS: a mixture of at least 11 Cos with different proportions of GlcNAc and GlcN), and fully acetylated (faCOS, mainly GlcNAc, (GlcNAc)2 and (GlcNAc)3) were produced. The anti-inflammatory activity of the three COS mixtures was studied by measuring their ability to reduce the level of TNF-α in stimulated LPS murine macrophages (RAW 264.7). Only fdCOS and faCOS were able to significantly reduce this factor [90,91]. The inhibition of NO secretion by COSs revealed that 10% acetylated COS inhibited NO secretion significantly more than those with 50% acetylation [92]. Citronellol grafted chitosan oligosaccharide derivatives have been produced to improve the anti-inflammatory activity of the oligosaccharides with degrees of substitution of 0.165, 0.199 and 0.182, respectively. In all cases, the derivatives showed better performance than the parent COS. These derivatives reduced the expression levels of TNF-α by promoting the secretion of IL-4 and IL-10 and inactivated the NF-κB signalling pathway via inhibiting the phosphorylation of p65, IKBα, and IKKβ [93]. 

Using the same chitosan as a starting material to produce chitooligosaccharides rendered samples with different anti-inflammatory behaviour. Chitooligosaccharides (5–10 kDa, DD: 87%) composed mainly of 42% fully deacetylated oligomers (A1-A3) plus 54% monoacetylated oligomers, produced by enzymatic degradation with chitosanase, attenuated the inflammation in lipopolysaccharide-induced mice and in RAW264.7 macrophages. On the contrary, chitooligosaccharides (5–10 kDa, DD: 89%) from a two-step preparation (chemical degradation followed by enzymatic degradation with chitosanase) were composed of 50% fully deacetylated oligomers plus 27% monoacetylated oligomers (A1-A3) promoted the inflammatory response in both in vivo and in vitro models [94]. This result shows how small differences in the COS mixture have a strong effect on the mixture behaviour.

## 5. Metallic Nanoparticles and Chitosan

Metallic nanoparticles are usually defined as particles of metal atoms with sizes ranging between 1 nm to a few hundred nanometres [95]. These particles exhibit optical, chemical, and electronic properties that differ from individual atoms or bulk materials. These unique properties are highly appreciated for different applications such as catalysis, photonics, or biomedicine [96].

**Figure 4:**
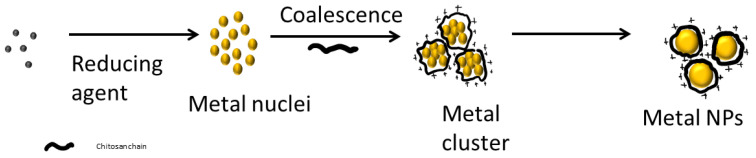
Scheme of metallic nanoparticle production and stabilization with chitosan.

Metallic nanoparticles can be prepared using myriad physical or chemical methods. Metal ions can be reduced using chemicals (NaBH_4_, vitamin C and others) [97,98], plant extracts (due to their phenolic compounds) [99], using polymers such as chondroitin sulphate or heparin [100,101], or using microorganisms containing specific enzymes such as nitrate reductase [102,103]. Other authors have proposed the use of sonochemical reduction [104], radiation [105], electrochemical reduction [106] or heat evaporation [107]. Once the formed metallic nanoparticles aggregate, the addition of stabilizers is needed [108] (Figure 4).

The synthesis of metallic nanoparticles using chitosan as a reducing agent and/or stabilizing agent is well described. Some authors have also proposed that chitosan plays a role in the control of nanoparticle nucleation, thus controlling nanoparticle size to some extent since metal concentration also affects the nanoparticle size [97,109].

The reducing and stabilizing properties of chitosan seems to be related to the presence of CH_2_OH, CHO, and NH_2_ groups in the polymeric chain. Changes in the molecular weight or deacetylation degrees not only alter the number of these reactive groups but also modify the interactions (hydrogen bonds, electrostatic interactions, or steric interactions) present in the system. 

In Table 4, some examples of the usage of chitosan in metallic nanoparticle synthesis are reviewed, including information about the molecules used as reducing agents, properties of the chitosan used when data are given, nanoparticle size, and morphology.

**Figure 5:**
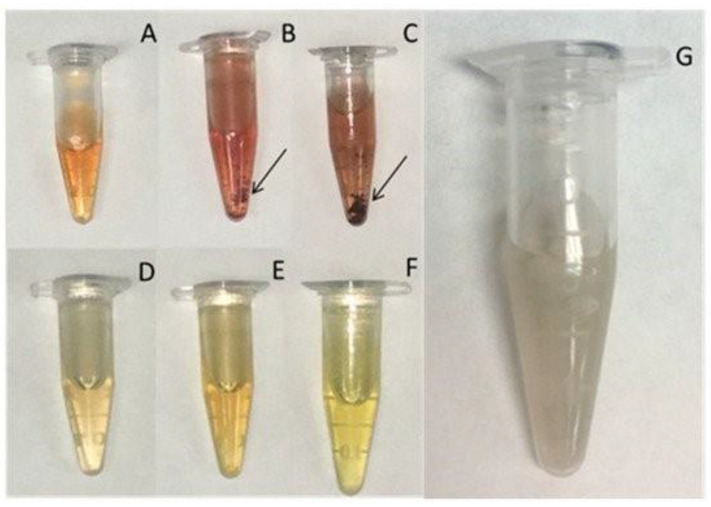
Visual evaluation of AgNP–polymer solutions after 5 h at 90 °C. (**A**) F1Q, (**B**) F2Q, (**C**) F3Q, (**D**) F1L, (**E**) F2L, (**F**) F3L, and (**G**) parent chitosan. Arrows indicate the presence of aggregates. © 2021 by the authors. Licensee MDPI, Basel, Switzerland (CC BY) license [123].

Data from Table 4 clearly show that the characteristics of the produced nanoparticles depend on the method used to produce the nanoparticles and the characteristics of the chitosan used to reduce and stabilize the metal ions. In general, due to the lack of a proper characterization of the chitosan samples and the variety of reaction conditions used it is very difficult to relate chitosan properties with the characteristics of the nanoparticles. Recently, the effect of chitosan Mw and acetylation degree on the preparation of AuNPs both as reducing and stabilizing agents has been analysed in detail [117]. The authors also took into consideration the effect of polymer and gold concentration, temperature, and reaction time. Their results showed that the chitosan acetylation degree and polymer concentration are the main parameters affecting the size and shape of the nanoparticles. Polymer molecular weight is related to the reductive efficiency since the reduction of the polymer size increases the amount of reducing sugars in the media. Our group has focused its research on the production of AgNPs using low molecular weight chitosan samples. As previously described in this review, the characteristics of these low molecular weight chitosan samples depend on the enzyme used to produce the samples. We hypothesised that samples with similar Mw and acetylation degrees may exhibit different behaviour due to the monomer pattern. Our results showed that pattern is a key parameter in the stabilization of the AgNPs, corroborating this hypothesis [123] A chitosan sample (538 kDa, DD 52%) with little ability to stabilize AgNPs was depolymerized with lysozyme (fraction L) and chitosanase (fraction Q) and the resulting reaction mixture was separated into three fractions by tangential ultrafiltration (fraction F1 (Mw > 30 kDa), fraction F2 (Mw 30–10 kDa), and fraction F3 (Mw 10–5 kDa). After depolymerization, an increase in the DD was observed with values between 62–74%). All fractions were able to reduce the silver ion, but relevant differences were observed in terms of stabilization (Figure 5). AgNPs produced with chitosan samples depolymerized with chitosanase (FQ2 and FQ3) were larger, poorly stabilized, and tended to form large aggregates visible with the naked eye. On the contrary, AgNPs produced with chitosan depolymerized with lysozyme were smaller and more stable in all cases. As the Mw of the fraction was reduced, the polydispersity was also lowered. After one month, the stability of the AgNPs was evaluated and results showed that AgNPs produced with the fractions F1Q and F1L were the most appropriate for nanoparticle stabilization.

The AgNPs produced with lysozyme fractions and the higher Mw fraction of chitosanase were tested in the catalytic reduction of TBO [124]. AgNPs produced through chitosan depolymerization with lysozyme showed better performance than the sample produced using chitosanase. Moreover, AgNPs produced with fraction F1L exhibited the best performance in the reaction. That is, the effect of the polymer pattern goes further than affecting optical properties and stability and differences in the catalytical behaviour was also observed. This difference is not due to the polymer, since control reactions showed that the polymeric fractions were not able to catalyse the reduction in TBO and therefore the effect is solely ascribed to the AgNPs.

## 6. Chitosan in Biocatalysis

**Figure 6:**
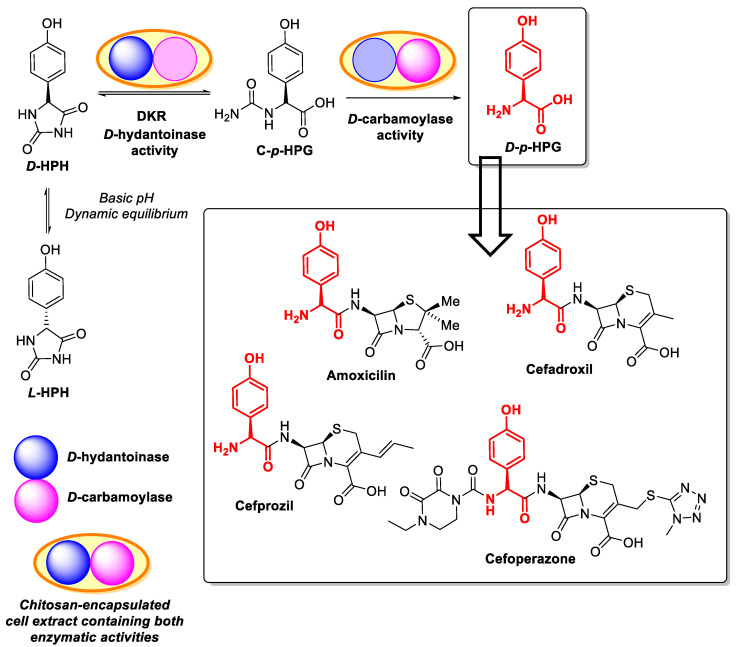
Schematic representation of the production of *p*-hydroxyphenylglycine (p-HPG) starting from a racemic mixture of *p*-hydroxyphenyl hydantoin (HPH) using a multi-enzyme system containing immobilized *D*-hydantoinase and *D*-carbamoylase.

The use of immobilized enzymes for catalysing chemo-, regio- and/or stereoselective chemical reactions is a very useful and well-known technique [125,126,127,128,129,130,131,132,133,134,135,136,137,138,139,140,141,142]. In this sense, the use of chitosan for immobilizing enzymes, either as a carrier for covalent linking or as an encapsulation vehicle, is well reported [143,144,145,146,147,148,149]. Our group described the production of enantiopure D-p-hydroxyphenylglycine (D-p-HPG, Figure 6) using a multi-enzyme system containing D-hydantoinase and D-carbamoylase encapsulated in chitosan-based materials [150,151,152,153].

*D-p*-HPG (or simply D-HPG, a D-amino acid) is a very useful chiral synthon, mainly used for the preparation of different semi-synthetic antibiotics, such as amoxicillin, cefadroxil, cefprozil, or cefoperazone [154,155,156] (Figure 6), but also anticancer drugs [157] and some heterocyclic compounds [158,159,160,161].

For preparing D-HPG, one of the most efficient processes is the so-called “hydantoinase process”, depicted in Figure 6. This cascade of enzymatic reactions, aiming to produce optically pure amino acids [162,163], requires an initial step catalyzed by a D-specific hydantoinase [E.C. 3.5.2.2.] to transform D-p-hydroxyphenyl hydantoin (D-HPH) into N-carbamoyl-D-p-hydroxyphenylglycine (C-p-HPG), which should be subsequently hydrolyzed by a second enzyme, a highly enantiospecific N-carbamoyl amino acid amidohydrolase (also termed D-carbamoylase; E.C.3.5.1.77), to furnish the free amino acid. One of the main features of the hydantoinase process derives from the spontaneous racemization of D-HPH at pH values higher than pH 8, caused by the acidic hydrogen at position 5 of the imidazolidine-2,4-dione ring, which allows for oxo-enol-tautomerism. This leads to a dynamic-kinetic resolution (DKR), allowing for the use of a mixture of L-and D-HPH as the initial substrate and a theoretical 100% conversion and 100% optically pure D-amino acid production (Figure 6).

Both enzymes have been reported to be present in different microorganisms, such as Agrobacterium sp., Pseudomonas sp., Arthrobacter crystallopoites, or Sinorhizobium morelense [151], and can be used either as whole cells, crude cell extracts, or purified enzymes (see Aranaz et al. [151] and references therein). If using isolated enzymes, immobilization is an excellent strategy for stabilizing the enzymatic cocktail due to the fact that D-hydantoinases are quite stable but D-carbamoylases display low thermostability and are prone to suffer oxidative degradations. In this sense, different protocols have been described (see Aranaz et al. [151] and references therein), and our group described how a multi-enzyme extract from Agrobacterium radiobacter rich in D-hydantoinase and N-carbamoyl-D-amino acid amidohydrolase was easily immobilized via adsorption on chitin and chitosan for its application in the synthesis of p-hydroxyphenylglycine [153]. In fact, this adsorption derivative on chitin showed higher activity compared to the covalent one, and much greater pH stability compared to the soluble multi-enzymatic extract; on the other hand, the adsorption derivative exhibited greater pH-stability in the pH range under study, showing higher activity at low temperatures. Anyhow, as the immobilized derivatives could not be properly reused, we developed a new strategy based on the encapsulation of a crude cell extract from the same microorganism, containing both enzymes, in alginate beads [164]. This biocatalyst could be reused six times in the presence of solid HPH particles in a stirred batch reactor without losing any activity until the beads started to burst. Anyhow, as these alginate-based catalysts showed low stability in calcium chelating buffers (i.e. phosphate buffers) and easy microbial contamination during storage at 4 °C, another immobilization matrix, alginate–chitosan polyelectrolyte complexes, was assessed [150,152]. Thus, alginate mixed chitosan capsules were prepared in one step (by simply dropping an alginate solution containing the extract into a chitosan solution containing calcium ions) or in a two-step process (preformed calcium–alginate capsules loaded with the crude cell extract were subsequently coated with chitosan). The encapsulation yields were around 60% and independent of the characteristics of the different chitosans used. However, p-HPG production was indeed affected by chitosan acylation degree D-D (the lower D-D, the lower p-HPG) but not by chitosan molecular weight. Generally speaking, the best biocatalyst allowed for a p-HPG production yield of around 60% without any significant protein release to the reaction media. Interestingly, this encapsulation procedure improved the stability of D-carbamoylase against oxidative damage during storage, particularly after freeze-drying. In addition, the alginate coated chitosan capsules could be reused eight times without enzymatic activity loss before D-carbamoylase started losing its activity and alginate–chitosan beads suffered burst problems contaminating the reaction.

**Figure 7:**
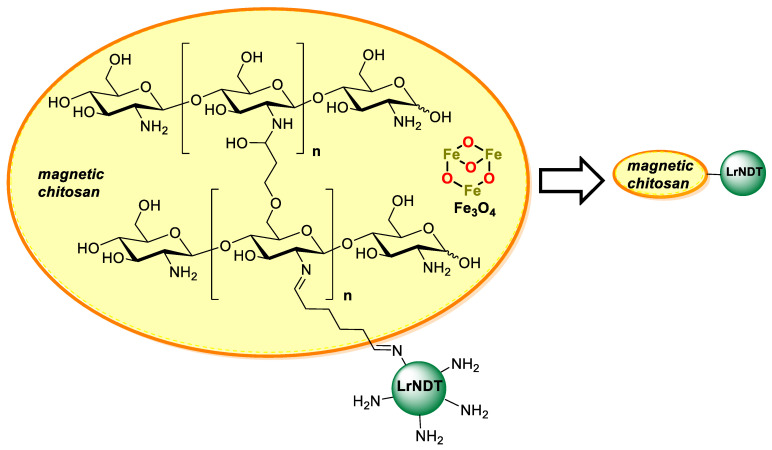
Schematic representation of the immobilization of a recombinant nucleoside 2′-deoxyribosyltransferase from *Lactobacillus reuteri* (LrNDT) on cross-linked magnetic chitosan beads. Adapted from Fernández-Lucas et al. [165].

In a collaboration with the group of Dr. Fernández-Lucas, we described the covalent immobilization of a recombinant nucleoside 2′-deoxyribosyltransferase from Lactobacillus reuteri (LrNDT) on cross-linked magnetic chitosan beads via epichlorohydrin activation under alkaline conditions, and subsequent incubation with glutaraldehyde [165], as schematized in Figure 7.

**Figure 8:**
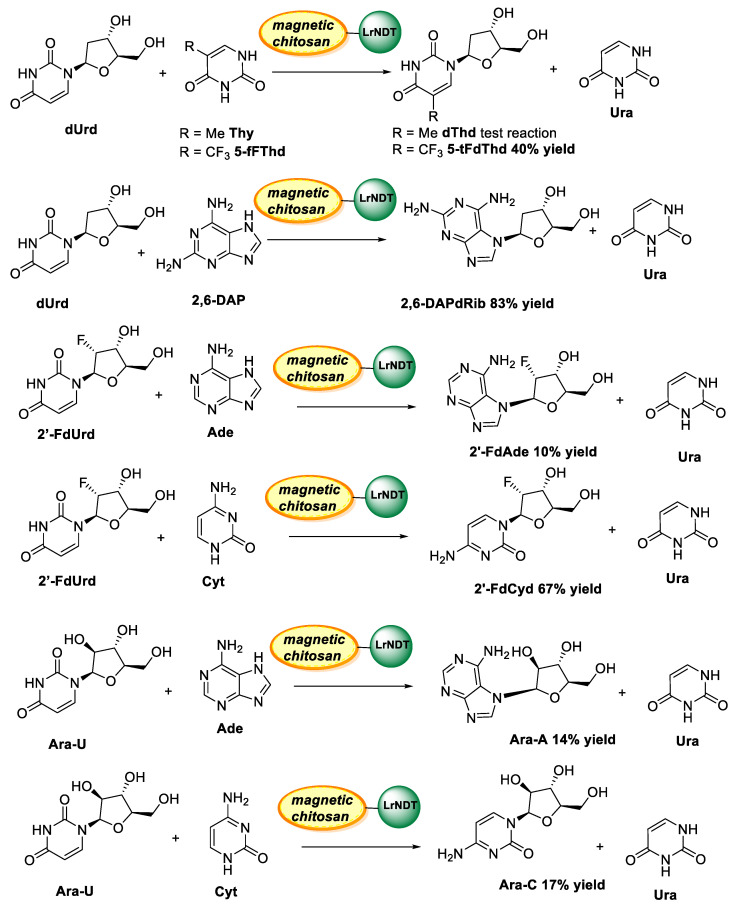
Synthesis of different natural and non-natural nucleosides using a recombinant nucleoside 2′-deoxyribosyltransferase from *Lactobacillus reuteri* (LrNDT) immobilized on cross-linked magnetic chitosan beads [165]. Commission on Biochemical Nomenclature: adenine (Ade), uracil (Ura), cytosine (Cyt), thymine (Thy), 2,6-diaminopurine (2,6-DAP), 5-trifluorothymine (5-tFThy), 2′-deoxyuridine (dUrd), 2′-deoxyadenosine (dAdo), 2′-deoxycytidine (dCyd), thymidine (dThd), 2,6-diaminopurine-2′-deoxyriboside (2,6-DAPdRib), 5-trifluorothymidine (5-tFdThd), 2′-fluoro-20-deoxyuridine (2′-FdUrd), 2′-fluoro-2′-deoxycitydine (2′-FdCyd), ara-uracil (ara-U), ara-adenine (ara-A).

Hence, by varying the amount of magnetite (Fe_3_O4) and epichlorohydrin (EPI), different macroscopic beads were prepared and fully characterized (by scanning electron microscopy, spin electron resonance (ESR), and vibrating sample magnetometry (VSM)) before being used as supports. Once activated with glutaraldehyde, the best support was chosen after assessment of immobilization yield and product yield using as a standard reaction for the synthesis of thymidine (dThd) from 2′-deoxyuridine (dUrd) and thymine (Thy), as depicted in Figure 7. Additionally, optimal conditions for chitooligosaccharides with the highest activity of immobilized LrNDT on magnetic chitosan were carried out using response surface methodology (RSM). Thus, the best-immobilized biocatalyst retained 50% of its maximal activity after 56.3 h at 60 °C and no lost activity was observed after storage at 40 °C for 144 h. Subsequently, this innovative immobilized biocatalyst was employed in the enzymatic synthesis of 2′-deoxyribonucleoside analogues and arabinosyl nucleosides such as vidarabine (ara-A) and cytarabine (ara-C), as depicted in Figure 8, leading to moderate or good yields at 2 h reaction time. Remarkably, the immobilized derivatives could be easily recovered and recycled for 30 consecutive batch reactions without any significant decrease in the catalytic activity in the synthesis of 2,6-diaminopurine-2′-deoxyriboside (2,6-DAPdRib) and 5-trifluorothymidine (5-tFThd).

## 7. Chitosan in Drug Delivery

Since the introduction of the first polymers in drug delivery, chitosan has shown superior biological and physiochemical properties for a wide variety of biomedical and industrial applications. The main feature of this biopolymer is its cationic character due to amino groups. These amino groups are also responsible for properties such as controlled drug release, mucoadhesion, in situ gelation, transfection, permeation enhancement, and efflux pump inhibitory properties [166]. Moreover, interest in this biomaterial due to its central nervous system (CNS) bio-medical implementation has increased because of its ability to cross the blood brain barrier (BBB) [167].

Therefore, chitosan is widely used in drug delivery due to its technological properties, which allow us to process the polymer in different ways (Table 5).

**Figure 9:**
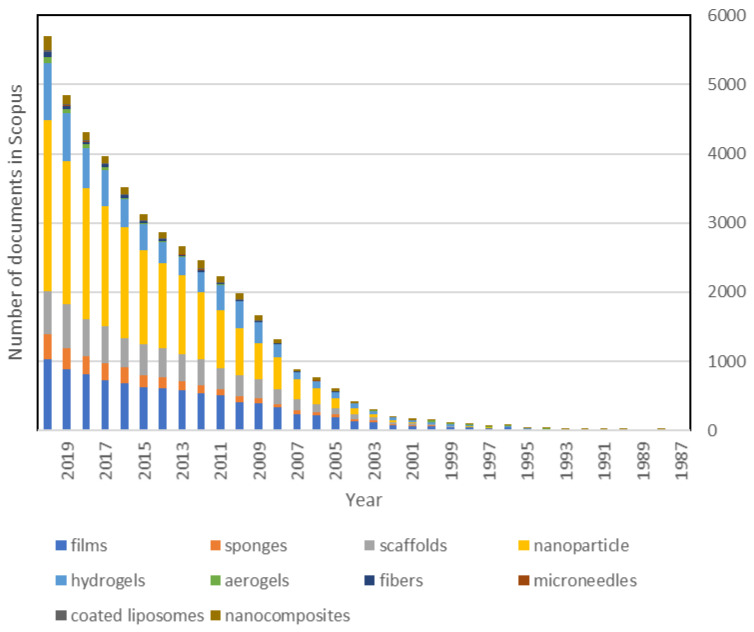
Publications about chitosan drug delivery in Scopus (1987–2020).

Initially, a chitosan salt (chitosan hydrochloride) was approved in 2002 by the Pharmacopeia. Chitosan was first introduced as an excipient into the European Pharmacopeia 6.0 and the 29th edition of the United States Pharmacopeia (USP) 34-NF almost ten years later. These monographs contain the assays and establish limits to be observed when the polymer is used as a pharmaceutical excipient [195,196]. The increase in the number of publications regarding the use of this polymer in drug delivery is shown in Figure 9 and reveals a strong increase since 2002 that is still maintained today. 

Chitosan films are easily produced by solvent-casting methodologies, but more complex systems can be produced by blending the polymer with others such as pectin [197] or by producing layer-by-layer films with negatively charged polymers like polyacid [198], poly (lactic-co-glycolic acid) [199] or polylactic [200], among others. Besides their safety, biocompatibility, and biodegradability, biopolymer-based films have been drawing increasing interest as excellent candidates not only as controlled-drug delivery systems but also as materials to produce contact lenses, wound dressings, and tissue engineering matrices.

Particulate chitosan-based systems (micro and nano systems) are widely used for the encapsulation of a large variety of molecules such as growth factors [178], antimicrobials [201], painkillers [202], anti-tumoral [203] or anti-inflammatory drugs [204]. 

Recently, chitosan has been used for the fabrication of microneedles (MNs) due to its film-forming ability, biodegradability, and biocompatibility, making it suitable for topical and transdermal drug delivery [188]. In particular, the use of chitosan MNs in vaccination is a hot topic of discussion [205,206,207]. The use of chitosan MNs in wound healing and point-of-care testing is revolutionary and gives hope of more useful developments in these areas. However, some drawbacks still need further investigation. The development of MNs devices with adequate mechanical strength to penetrate the skin without causing pain and skin damage and the development of efficient methods for their sterilization remain challenging [208].

**Figure 10:**
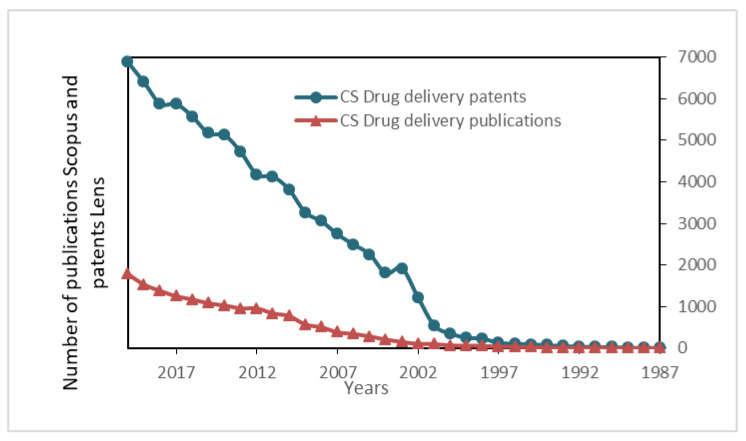
Publications about chitosan drug delivery in Scopus and patents in Lens (1987–2020).

A comparison of the number of publications containing “Chitosan + drug delivery” in Scopus and patents in Lens portal (free, open patent, and scholarly search) is shown in Figure 10. As observed, the number of patents is almost four times the number of publications, showing the increasing application of this polymer in the drug delivery field. An interesting article by Kurakula and Raghavendra summarizes the chitosan biomedical trends and the related patents [209].

## 8. Conclusions and Prognosis

**Figure 11:**
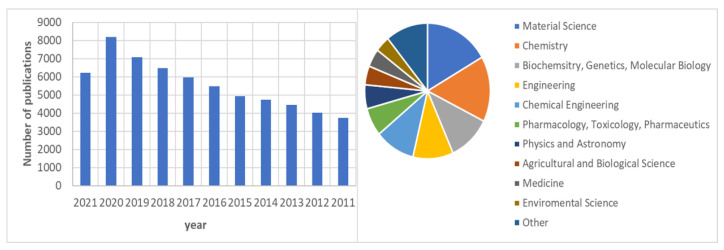
Number of publications and distribution by area in the period 2011–2021. Search of chitosan word in Scopus (abstract, title, keywords).

Chitosan and its derivatives have been used in a myriad of applications for a long time. The potential interest of these polymers is clear when observing the number of articles and patents that appear every year and the growing market perspective. In some of these applications such as agriculture or the food industry, the use of chitosan in the market is well established. The use of chitosan has extended to a large number of research areas from Materials Science to Arts and the Humanities (Figure 11).

However, chitosan potentiality is somehow hindered by the inconsistency in the research data and the lack of knowledge in the ultimate mechanism underlying the properties of chitosan. Between 2011–2020, the number of publications on chitosan has displayed a steady growth. In 2021, a drop is observed, which is ascribed in part to the large number of reviews published in 2020, probably due to the COVID-19 pandemic, which has affected normal laboratory work worldwide. Regardless, we consider that this growth will continue in the following years, driven by the strong effort that has been carried out by the Chitin Science Scientific Community in the systematic research on this polymer. In fact, its approval by different agencies has boosted the interest in this polymer both by the industrial and scientific communities.

Chitosan specifications are ultimately related to its final application. Thus, high quality chitosans with low heavy metal and low endotoxin contents are required for biomedical and pharmaceutical uses. Moreover, strict control of production is needed to avoid uncontrollable hydrolysis and chemical modifications during polymer isolation. Therefore, chitosan production is not a trivial issue. To date, chitosan production cannot be considered fully sustainable due to the large amount of acid and basic reagents needed and the high temperatures required. Unfortunately, biotechnological processes using biocatalysts are currently limited to the laboratory scale so that implementation of these greener processes at a large scale is certainly one of the milestones we want to see being achieved in the next decade.

## Figures and Tables

**Table 1:** The main properties of chitin and chitosan.

Property/Activity	Reference
Mucoadhesive	[5,6]
Anti-inflammatory	[7]
Antioxidant	[8]
Antimicrobial	[9]
Antifungal	[10]
Antihyperglycemic	[11]
Antitumoral	[7,8,9,10,11,12]
Wound healing	[13]

**Table 2:** The main products produced in the enzymatic degradation of chitosan.

Enzyme	Main Product
Chitosanase	Oligomers DP 2–3
Hemicellulase	Dimers, trimers, tetramers, pentamers and hexamers
Pepsine	Glucosamine, N-acetylglucosamine oligomers with DP 2–6
Pronase	4–10 kDa
Papain	Glucosamine, N-acetylglucosamine oligomers with DP 2–6
Lipase	High DP

DP: depolymerization degree.

**Table 3:** Antimicrobial and antifungal activity of chitosan.

System	Target	Inhibition	References
Chitosan	*Aeromonas hydrophila* *Edwardsiella ictalurid* *Flavobacterium columnare*	Complete 0.4% (E I, F C) 0.8% (A. H)	[50]
Chitosan	*Candida albicans* Gram-positive bacteria (such as *Bacillus cereus*, *S. aureus*, *Bacillus megaterium*, *Lactobacillus plantarum*, *Listeria monocytogenes*, *Lactobacillus brevis*, and *Lactobacillus bulgaricus)* Gram-negative bacteria (such as *Salmonella typhimurium*, *E. coli, Pseudomonas aeruginosa*, *Pseudomonas fluorescens*, *Vibrio parahaemolyticus*, *Enterobacter aerogenes*, and *Vibrio cholera)*	Strong and safe effect	[51,52]
Chitosan hydrochloride Carboxymethyl chitosan Chitosan oligosaccharide *N*-acetyl-*D*-glucosamine	*Candida krusei*, *C. albicans*, *C. glabrata*	No effect: chitosan oligosaccharide and *N*-acetyl-*D*-glucosamine. Weak effect: Carboxymethyl chitosan. Strong effect: Chitosan hydrochlorides.	[53]
Chitosan wound dressing	*P. aeruginosa*, *B. cereus*, *L. monocytogenes*	Strong effect: wound management due to their antimicrobial nature, ability to accelerate wound contraction and healing, haemostatic and analgesic	[54,55,56,57]
Chitosan sponges	*S. aureus, E. coli*		[58,59]
Chitosan microparticles and nanoparticles	*E. coli*, *Vibrio cholerae*, *S. enterica*, *Streptococcus uberis*, *S. uberis*, *S. enterica*, *K. pneumonia*, *S. aureus*, *V. cholerae, Salmonella choleraesuis*, *S. typhimurium*	Strong effect	[60,61,62]

**Table 4:** Metallic nanoparticle based on chitosan.

Metal	Reducing Agent	Stabilizer Chitosan Mw and DD	NPs Size	Morphology	Ref.
Palladium	Ascorbic acid	Cs 180 kDa, 75–85% DD	5–20	Spherical	[98]
	Ascorbic acid	Cs 50 to 190 kDa, 75–85%	50–70	Flower-spherical	[110]
	Ascorbic acid	Cs, 50 to 190 kDa, 75–85%	30–150	Flower	[111]
	Ascorbic acid	TMCs 20 kDa	55–120	Spherical	[112]
	NaBH_4_	Cs, 400 kDa DD 100%	nd	nd	[109]
	NaBH_4_	Cs, (~400 kDa)	2	Spherical	[113]
	MeOH	Cs, (~400 kDa)	2–5	Spherical, large aggregate (Pd:MeOH 10:1)	[113]
	Hydrazine N_2_H_4_	Cs, (~400 kDa)	20 *	Highly aggregate	[113]
Platinum	NaBH_4_	Cs, 400 kDa DD 100%	2–5	spherical	[109]
	NaBH_4_	Cs, (~400 kDa)	2–3	spherical	[113]
	MeOH	Cs, (~400 kDa)	2	spherical	[113]
	Hydrazine N_2_H_4_	Cs, (~400 kDa)	17–25 *	aggregates	[113]
Gold	Cs, 1278 kDa	Cs, 1278 kDa	16		[114]
	Cs 817 KDa	Cs, 817 KDa	5	Spherical	[115]
	NaBH_4_	Cs, 400 kDa DD 100%			[109]
	Cs DD > 85%; >200,000 cps	Cs, DD > 85%; >200,000	5–20	Spherical	[101]
	NaBH_4_	Cs n.c.	6–20	Spherical; polyhedral	[97]
	COS 5 kDa	COS 5 kDa	7–15	Spherical	[116]
	Cs,	Cs, DD 53–95%, Mw 2.6–490 kDa	5–200 nm	Spherical, triangles, polyhedral	[117]
Silver	Cs	Cs 1240 kDa, DA 0.13	10–150	Spherical Triangles in long storage	[118]
	Cs	Cs, high Mw, DA 0.25	5	Spherical	[119]
	Cs DD > 85%; >200,000 cps	Cs DD > 85%; >200,000 cps	20–200	Spherical, fractal	[101]
	Ascorbic acid	Cs 180 kDa, 75–85% DD	5–20	Spherical	[98]
	NaBH_4_	Cs 400 kDa DD 100%	30–200	Spherical clusters	[109]
	Gamma radiation	Cs n.c.	4–5	Spherical	[101]
	Cs n.c.	Cs n.c.	10–60	Spherical	[120]
	Ascorbic acid/Cs 1278 kDa	Cs 1278 kDa	8		[114]
	Cs n.c.	Cs n.c.			[121]
	Cs	Cs (50–190 kDa DD 75–85%)		Fractal patterns	[122]

Cs: chitosan; TMCs: trimethyl chitosan; n.c.: non-characterized; nd: non-determined; * aggregate size.

**Table 5:** Some examples of chitosan presentations in drug delivery.

Presentation	References
Films	[168,169,170,171]
Sponges	[172,173]
Scaffolds	[174,175]
Nanoparticles	[176]
Microspheres	[177,178,179]
Hydrogels	[180,181,182]
Aerogels	[183,184,185]
Fibers	[186,187]
Microneedles	[188,189]
Coated Liposomes	[190,191]
Nanocomposites	[192,193]
Composites	[194]

## Data Availability

Not applicable.
